# Arterial Hypertension and Health-Related Quality of Life

**DOI:** 10.3389/fpsyt.2017.00270

**Published:** 2017-12-04

**Authors:** Vasiliki Katsi, Manolis S. Kallistratos, Konstantinos Kontoangelos, Pavlos Sakkas, Kyriakos Souliotis, Costas Tsioufis, Petros Nihoyannopoulos, George N. Papadimitriou, Dimitris Tousoulis

**Affiliations:** ^1^Cardiology Department, Hippokration Hospital, Athens, Greece; ^2^Cardiology Department, Asklepeion General Hospital, Athens, Greece; ^3^1st Department of Psychiatry, National and Kapodistrian University of Athens, Eginition Hospital, Athens, Greece; ^4^University of Peloponnese, Corinth, Greece; ^5^1st Department of Cardiology, National and Kapodistrian University of Athens, Athens, Greece

**Keywords:** arterial hypertension, depression, SF36, awareness, quality of life

## Abstract

**Purpose:**

To investigate the effect of awareness of arterial hypertension on quality of life in hypertensive patients in Greece.

**Materials and methods:**

This was a prospective observational study that included 189 aware hypertensive patients on treatment with antihypertensive therapy. Patients were ambulatory men or women ≥18 years old, with diagnosed essential hypertension. The administration and fulfillment of the questionnaires was given at the outpatient hypertensive clinic starting with the SF-36 and continuing with the BDI-I test.

**Results:**

The mean BDI score was 9.9 ± 6.9, and 58, 25, 8.9, and 7.3% were identified as without, with minimal, moderate, and 0.8% with severe depression, respectively. The mean score for physical component summary (PCS-36) was 48.9 ± 7.6, and the mean score for mental component summary (MCS-36) was 46.0 ± 10.6. The stage of hypertension was not an independent predictor for any of the SF-36 dimensions. Dippers had not different levels of health-related quality of life (HRQOL) as compared with non-dippers. LV hypertrophy was associated with lower scores on bodily pain (*p* < 0.05) and kidney failure was associated with lower scores on general health perception (*p* < 0.05). Female gender, increased age, and the presence of COPD were independently associated with lower physical and mental health scores (*p* < 0.05). Score on BDI independently correlated with all dimensions of SF-36, indicating that greater depression levels are associated with lower levels of HRQOL.

**Conclusion:**

The stage as well as awareness of arterial hypertension does not affect physical and mental health. The fact that arterial hypertension *per se* is not a symptomatic disease may explain these results at least in patients with uncomplicated hypertension.

## Introduction

Several studies affirmed that awareness of having a chronic disease may affect health-related quality of life (HRQOL) and that it may have greater impact on mental health than having the disease itself (1–4). Arterial hypertension is a chronic disease affecting 30–45% of the general population, with a steep increase with age (5). Although it is usually asymptomatic, several studies affirm that arterial hypertension may influence HRQOL. According to the study by Mena-Martin et al. (6), individuals who are aware of being hypertensive have a poorer quality of life (QOL) concerning general health, physical functioning, vitality, and mental health than those who are not aware. Moreover, in a recent study from Cyprus (7), arterial hypertension was related to anxiety and depression. There are studies affirming that arterial hypertension represents a risk factor for the development of depression (8) and *vice versa*, that is, depression increases the risk of hypertension (9). However, there are studies affirming that arterial hypertension has little or no effect on HRQOL. In a large multicenter study that included 24,936 participants, the difference between hypertensive and normotensive patients in HRQOL in both mental component summary (MCS) and physical component summary (PCS) Short-Form Health Survey (SF)-36 components was of small magnitude (1). Likewise, in a similar study with 466 participants from Spain, the authors did not find differences in the HRQOL component scores (6). Thus, we thought to investigate the effect of arterial hypertension on QOL in patients who are aware of being hypertensive in Greece.

## Materials and Methods

This was a prospective observational study that included 189 hypertensive patients on treatment with antihypertensive therapy. The first patient was enrolled on October 1, 2016 and the date of the last visit of the last patient was November 21, 2016. Patients were ambulatory men or women ≥18 years old, diagnosed with hypertension. Patients had to be on treatment with antihypertensive agents for at least 3 months in order to be aware of arterial hypertension. The diagnosis of hypertension was established combining office and 24-h ambulatory blood pressure measurements according to the current ESH guidelines (5). Exclusion criteria included secondary hypertension; serious end-stage diseases (cancer or serious liver, respiratory, heart, or renal insufficiency); severe neuropsychiatric diseases; cerebrovascular events with serious residual neurologic deficit; and pregnancy, lactation, or desire to become pregnant, having a psychiatrist disorder or being under relevant treatment for such disorder. Demographic characteristics, cardiovascular parameters, coexisting risk factors for hypertension, comorbidities, and concomitant medication were identified at inclusion. Systolic blood pressure (SBP) and diastolic blood pressure (DBP) were measured according to ESH/ESC guidelines for the management and treatment of hypertension (5). All patients underwent 24-h ambulatory blood pressure measurement (in order to assess the dipping status), cardiac, and carotid ultrasounds. This study was designed in line with the recommendations of the latest Declaration of Helsinki (2013), the ISPE Guidelines for Good Pharmacoepidemiology Practice, and the ICH Guidelines for Good Clinical Practice, and was approved by the appropriate Ethics Committee (Scientific Councils) of participating hospital departments. All patients gave informed consent prior to their inclusion in the study.

### Assessment of HRQOL

Health-related quality of life is one of the several variables commonly studied in the field of medical outcomes research. It encompasses a wide range of human experience, including functioning and subjective responses to illness. Contemporary interpretations of HRQOL are based on the World Health Organization’s definition of health as a state of complete physical, mental, and social well-being and not merely the absence of disease. HRQOL was assessed with the use of a self-administered questionnaire, the SF-36 (Greek standard version 1.0). The SF-36 is a generic measure of health status, which is not age or disease specific and consists of eight domains: physical functioning (PF), role physical (RP), bodily pain (BP), general health perception (GH), vitality (VT), social functioning (SF), role emotional (RE), and mental health (MH). Scores of PCS-36, MCS-36, and the eight domains were calculated from raw data, according to the SF-36 manual. The domain scores were scale data of 0–100 and the summaries were deviation scores of mean 50. Missing values were treated according to procedures suggested in the SF-36 manual (10).

### Assessment of Depressive Symptomatology

The Beck Depression Inventory (BDI-I) (11) was used to assess the depressive symptomatology. The BDI-I is a 21-question multiple-choice self-report inventory that assesses cognitive, affective, and somatic depressive symptoms occurred over the past week. The 21 items are scored on a 0–3 scale, yielding a score range of 0–63 with higher scores indicating greater depression severity. The BDI score is not a diagnostic tool to assess major depressive disorder. In this study, we used the Greek BDI version, which was validated and applied to patients with arterial hypertension, cancer, and neurological disorders (12, 13).

The administration and fulfillment of the questionnaires was given at the outpatient hypertensive clinic starting with the SF-36 and continuing with the BDI-I test. All the patients were clinically evaluated by psychiatrists regarding their psychiatric history (depressive symptoms during the past, or hypomanic and delusional symptoms). Family history of psychiatric disorder was examined. All the patients filled the questionnaires at the hospital under the supervision of psychiatrists.

### Statistical Analysis

Continuous variables are presented with mean and standard deviation (SD). Quantitative variables are presented with absolute and relative frequencies. Linear regression analyses were used in order to find the factors independently associated with HRQOL dimensions and the Beck Depression Inventory (BDI) score. Adjusted regression coefficients (β) with SEs were reported from the results of the linear regression analyses. All *p* values reported are two-tailed, the significance level was set at 0.05 and analyses were conducted with the SPSS statistical package (Version 19.0).

## Results

The sample consisted of 189 hypertensive patients (85 men and 104 women) with mean age of 52.9 years (SD = 11.9 years). A total of 47.1% of the sample were smokers, while 3.7% of the participants had chronic obstructive pulmonary disease (COPD), 11.6% had diabetes, and 64% had hypercholesterolemia. Renal impairment and stroke were reported from 4.9 to 3.2% of the participants, respectively. Concerning stage of hypertension, 55.6% of the sample was at stage I, 36% at stage II, and 8.5% at stage III. Patient’s characteristics are presented in Table 1.

**Table 1:** Patient’s characteristics.

	*N* (%)
Sex	
Men	85 (45.0)
Women	104 (55.0)
Age, mean (SD)	52.9 (11.9)
Family status	
Unmarried	46 (24.3)
Married	134 (70.9)
Divorced	8 (4.2)
Widowed	1 (0.5)
Smoking	89 (47.1)
COPD	7 (3.7)
Diabetes	22 (11.6)
Hypercholesterolemia	121 (64)
Stage of hypertension	
I	105 (55.6)
II	68 (36.0)
III	16 (8.5)
DIPPERS	126 (66.7)
LV hypertrophy	72 (48.0)
Renal impairment	9 (4.9)
Stroke	6 (3.2)
Carotid disease	58 (42.0)

*COPD, chronic obstructive pulmonary disease; LV, left ventricle*.

**Figure 1:**
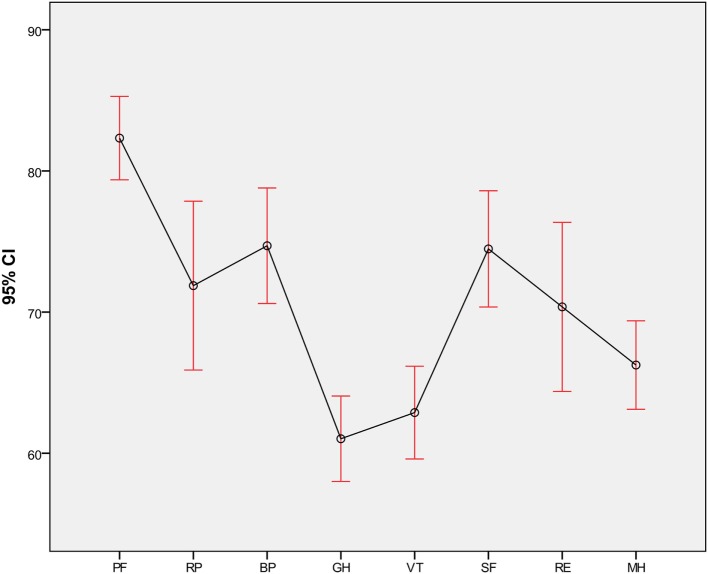
Mean values for SF-36 dimensions. PF, physical functioning; RP, role physical; BP, bodily pain; GH, general health perception; VT, vitality; SF, social functioning; RE, role emotional; MH, mental health.

The mean BDI score was 9.9 (SD = 6.9), and 58% were identified without depression, 25% with minimal depression, 8.9% with mild depression, 7.3% with moderate depression, and 0.8% with severe depression. The mean scores for SF-36 dimensions are shown in Figure 1. The mean score for PCS-36 was 48.9 (SD = 7.6), and the mean score for MCS-36 was 46.0 (SD = 10.6).

Table 2 shows multiple linear regression analysis results with dependent variable, the BDI score. Higher levels of depression were found in women and in those with diabetes. Dippers reported lower levels of depression.

**Table 2:** Multiple linear regression analysis results with dependent variable: the BDI score.

	β (SE)^a^	*p*
Sex		
Men	0.00^b^	
Women	6.38 (1.84)	**0.001**
Age	0.08 (0.08)	0.321
Married		
No	0.00	
Yes	−2.16 (2.13)	0.315
Smoking	1.64 (1.78)	0.362
COPD	−5.8 (6.9)	0.403
Diabetes	6.91 (2.69)	**0.013**
Hypercholesterolemia	2.37 (1.95)	0.230
Duration	−0.01 (0.03)	0.567
Stage		
I/II	0.00	
III	0.05 (3.27)	0.987
Dippers	−3.69 (1.84)	**0.050**
LV hypertrophy	0.78 (1.41)	0.583
Renal impairment	1.28 (4.85)	0.793
Stroke	3.38 (4.37)	0.442
Carotid disease	−0.6 (1.95)	0.759

*^a^Regression coefficient (SE)*.

*^b^Reference category*.

*COPD, chronic obstructive pulmonary disease; LV, left ventricle; BDI, Beck Depression Inventory score*.

**Figure 2:**
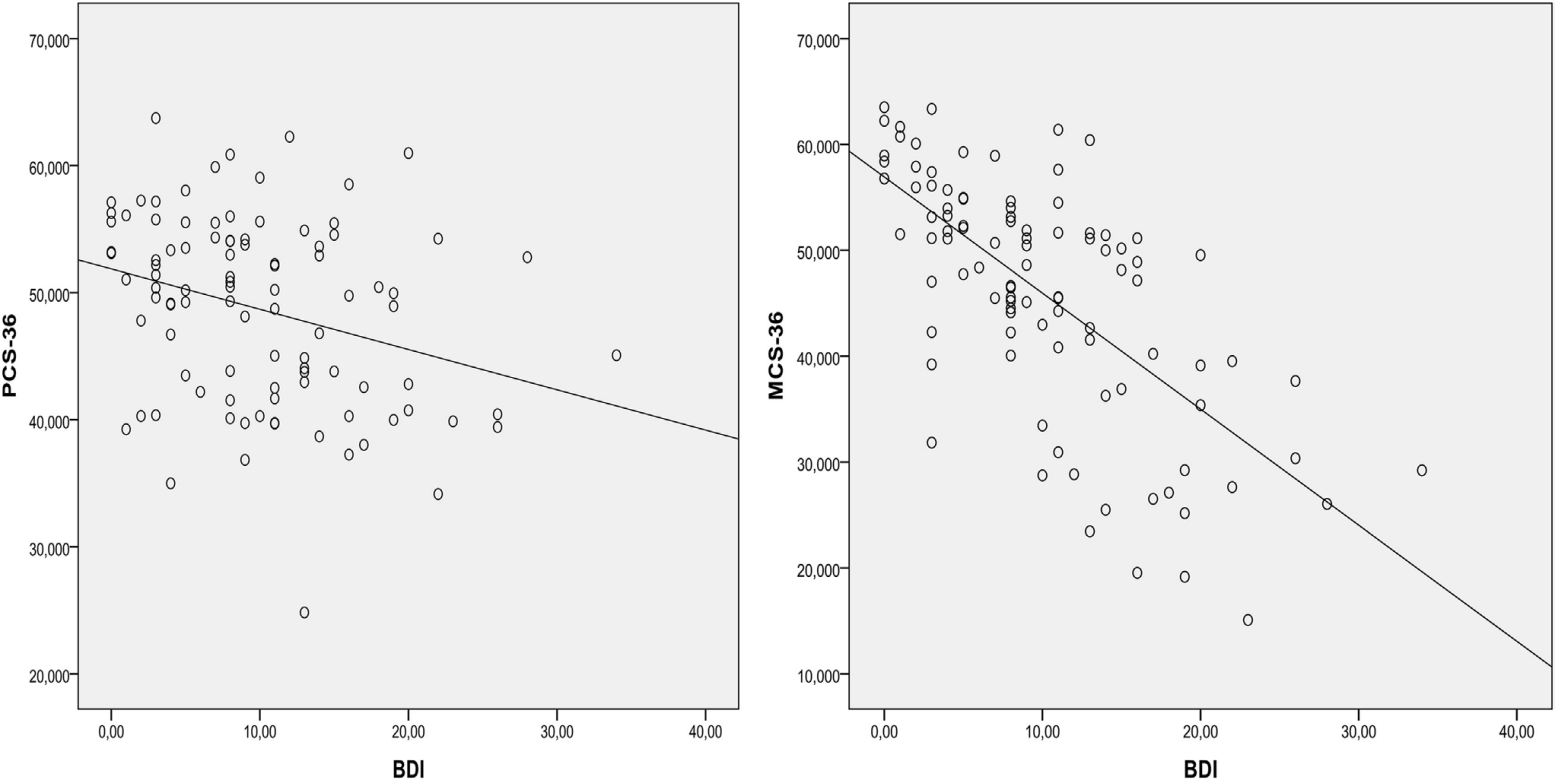
Scatter plots of BDI with PCS-36 and MCS-36. BDI, Beck Depression Inventory score; PCS 36, physical component summary; MCS 36, mental component summary.

The results of multiple linear regression analysis for dimensions of SF-36 related to physical and mental health are presented in Tables 3 and 4, respectively. Women had lower scores on BP, PCS-36, SF, RE, VT, and MCS-36. Increased age was independently associated with lower scores on PF and RE. The presence of COPD was associated with lower scores on PF, RP, and PCS-36, while the presence of diabetes was associated with lower scores on MH and MCS-36. Stage of hypertension was not an independent predictor for any of the SF-36 dimensions. Also, dippers had not different levels of HRQOL as compared with non-dippers. LV hypertrophy was associated with lower scores on BP, and kidney failure was associated with lower scores on GH, RE, MH, VT, and MCS-36. Score on BDI was independently correlated with all dimensions of SF-36, indicating that greater depression levels are associated with lower levels of HRQOL. Scatter plots of BDI with PCS-36 and MCS-36 are shown in Figure 2.

**Table 3:** Results of multiple linear regression analysis for dimensions of SF-36 related to physical health.

	PF		RP		BP		GH		PCS-36	
	β (SE)^a^	*p*	β (SE)^a^	*p*	β (SE)^a^	*p*	β (SE)^a^	*p*	(SE)^a^	*p*
Sex										
Men	0.00^b^		0.00^b^		0.00^b^		0.00^b^		0.00^b^	
Women	−7.91 (5.24)	0.138	−20.71 (10.58)	0.056	−13.4 (6.75)	0.050	−7.6 (6.16)	0.224	−5.42 (2.56)	0.041
Age	−0.6 (0.21)	0.006	−0.51 (0.44)	0.253	−0.26 (0.28)	0.347	0.38 (0.25)	0.132	−0.19 (0.1)	0.076
Married										
No	0.00		0.00		0.00		0.00		0.00	
Yes	1.59 (5.54)	0.776	−11.01 (11.12)	0.327	−6.6 (7.13)	0.359	−2.09 (6.25)	0.740	0.39 (2.53)	0.878
Smoking	−5.46 (4.62)	0.243	−4.3 (9.45)	0.651	−0.26 (5.96)	0.965	−7.34 (5.73)	0.207	−1.44 (2.36)	0.547
COPD	−21.97 (7.82)	0.006	−36.67 (15.11)	0.016	−19.62 (10.03)	0.052	−14.47 (8.37)	0.086	−14.87 (3.36)	<0.001
Diabetes	−3.35 (7.26)	0.647	2.75 (14.72)	0.853	6.62 (9.47)	0.488	4.68 (8.83)	0.598	1.4 (3.54)	0.695
Hypercholesterolemia	6.46 (4.96)	0.199	−7.36 (5.5)	0.182	−7.56 (3.56)	0.035	−0.04 (5.74)	0.995	2.51 (2.31)	0.285
Duration	−0.02 (0.07)	0.759	−0.03 (0.14)	0.797	−0.14 (0.08)	0.098	−0.01 (0.08)	0.867	−0.03 (0.03)	0.403
Stage										
I/II	0.00		0.00		0.00		0.00		0.00	
III	−7.62 (8.91)	0.397	−20.39 (19.17)	0.293	−17.25 (10.85)	0.118	−5.67 (11.85)	0.635	−4.78 (4.88)	0.333
Dipper	1.7 (5.04)	0.738	2.45 (10.05)	0.808	10.89 (6.33)	0.092	2.46 (6.21)	0.694	4.28 (2.6)	0.107
LV hypertrophy	0.19 (3.19)	0.954	5.7 (6.07)	0.350	−9.12 (3.99)	0.024	2.81 (3.2)	0.381	1.8 (1.36)	0.189
Renal impairment	−1.41 (11.88)	0.906	−2.23 (25.03)	0.929	−5.69 (16.07)	0.725	−13.7 (6.74)	0.044	−2.22 (5.42)	0.685
Stroke	−1.6 (11.13)	0.886	12.14 (29.24)	0.680	−10.49 (14.58)	0.475	12.6 (13.04)	0.340	−3.73 (6.62)	0.576
Carotid disease	−0.79 (5.04)	0.875	−1.38 (10.55)	0.897	2.96 (6.47)	0.650	3.75 (6.07)	0.540	1.64 (2.61)	0.533
BDI score	−0.98 (0.36)	0.009	−1.51 (0.74)	0.046	−1.28 (0.46)	0.008	−1.26 (0.42)	0.005	−0.32 (0.10)	0.002

*^a^Regression coefficient (SE)*.

*^b^Reference category*.

*PF, physical functioning; RP, role physical; BP, bodily pain; GH, general health perception; PCS 36, physical component summary; COPD, chronic obstructive pulmonary disease; LV, left ventricle; BDI, Beck Depression Inventory score*.

**Table 4:** Results of multiple linear regression analysis for dimensions of SF-36 related to mental health.

	SF		RE		MH		VT		MCS-36	
	β (SE)^a^	*p*	β (SE)^a^	*p*	β (SE)^a^	*p*	β (SE)^a^	*p*	β (SE)^a^	*p*
Sex										
Men	0.00^b^		0.00^b^		0.00^b^		0.00^b^		0.00^b^	
Women	−17.85 (3.38)	<0.001	10.64 (5.40)	0.043	1.9 (4.4)	0.668	−11.44 (2.87)	<0.001	3.82 (1.74)	0.030
Age	−0.15 (0.24)	0.546	−0.49 (0.23)	0.033	0.24 (0.18)	0.187	0.27 (0.2)	0.181	−0.01 (0.11)	0.940
Married										
No	0.00		0.00		0.00		0.00		0.00	
Yes	−4.06 (6.23)	0.518	−18.57 (11.51)	0.113	−2.37 (3.06)	0.439	−7.03 (5.2)	0.183	−0.31 (1.96)	0.876
Smoking	−2.52 (3.42)	0.461	−9.8 (9.63)	0.314	−6.39 (3.85)	0.103	−6.56 (4.38)	0.141	−2.41 (1.78)	0.179
COPD	−8.28 (9.85)	0.402	−11.33 (15.15)	0.456	4.83 (8.02)	0.548	−9.02 (8.28)	0.278	2.61 (4.71)	0.580
Diabetes	−2.37 (8.27)	0.776	−7.71 (15.25)	0.615	−12.47 (4.29)	0.004	−1.34 (6.82)	0.845	−6.26 (2.78)	0.026
Hypercholesterolemia	−8.99 (5.71)	0.122	−15.57 (10.55)	0.147	4.04 (4.23)	0.345	3.51 (4.81)	0.469	2.47 (2.53)	0.334
Duration	−0.09 (0.07)	0.220	0.07 (0.14)	0.625	−0.08 (0.05)	0.171	−0.01 (0.06)	0.884	0.01 (0.03)	0.985
Stage										
I/II	0.00		0.00		0.00		0.00		0.00	
III	−0.81 (9.47)	0.932	−11.24 (19.34)	0.564	−11.89 (6.98)	0.095	−15.85 (7.88)	0.070	−6.3 (5.33)	0.245
Dipper	0 (5.53)	1.000	6.41 (10.4)	0.540	4.27 (4.19)	0.313	2.89 (4.83)	0.553	5.31 (2.84)	0.069
LV hypertrophy	1.98 (3.94)	0.616	−8.22 (6.05)	0.176	1.83 (3.2)	0.568	−4.51 (3.34)	0.180	1.14 (1.91)	0.552
Renal impairment	−7.66 (14.03)	0.588	−27.92 (13.21)	0.036	−14.41 (6.69)	0.033	20.09 (7.59)	0.009	−10.7 (4.03)	0.009
Stroke	3.84 (12.72)	0.764	−2.21 (23.79)	0.926	−7.49 (9.32)	0.426	−0.1 (10.49)	0.992	−6.63 (7.23)	0.365
Carotid disease	1.34 (5.65)	0.813	8.72 (10.75)	0.422	−0.98 (4.28)	0.821	0.16 (4.97)	0.975	1.95 (2.85)	0.498
BDI score	−1.76 (0.4)	<0.001	−2.35 (0.75)	0.003	−1.8 (0.29)	<0.001	−1.53 (0.33)	<0.001	−0.82 (0.18)	<0.001

*^a^Regression coefficient (SE)*.

*^b^Reference category*.

*SF, social functioning; RE, role emotional; MH, mental health; VT, vitality; MCS 36, mental component summary; COPD, chronic obstructive pulmonary disease; LV, left ventricle; BDI, Beck Depression Inventory score*.

## Discussion

Unfortunately, there are only few studies on assessing how awareness of arterial hypertension may influence HRQOL. Although factors such as gender, age, and diseases such as diabetes mellitus, COPD, and renal impairment seem to affect physical and mental health, the presence of arterial hypertension did not affect these parameters. It is well known that gender influences physical and mental health, since female gender more often reports feelings of dissatisfaction and frustration, which mainly influence the psychological domain (14). Several studies report worse QOL in the female sex regarding the domain of functional capacity, pain, limitation due to emotional aspects, and mental health of SF-36 (15). In addition, men similar to women are generally better able to tolerate chronic diseases without being emotionally affected (16). Likewise, the presence of diseases, such as diabetes mellitus, COPD, renal impairment, negatively influence ordinary activities and daily routine but significantly influence physical and mental health (16–19). In patients with COPD, dyspnea represents the main reason of impaired functional status, and the severity of dyspnea significantly affects physical and mental health (17). In addition, the presence of diabetes mellitus significantly affects the QOL (18, 19), since treatments are burdensome and the complications can be debilitating and life threatening (18). In a study that enrolled 240 diabetic patients, all SF-36 scales were predicted by severity and number of diabetes complications (18). Finally, patients with chronic kidney disease seem to have poorer HRQOL than that of the general population (20). Several factors such as anemia, associated diseases, and early treatment by a nephrologist appear to have an impact on the QOL of these patients (20–22). In this study, the presence as well as the stage of arterial hypertension did not affect physical and mental health. This is in accordance with other studies affirming that arterial hypertension has little or no effect on HRQOL. In a large multicenter study that included 24,936 participants, the difference between hypertensive and normotensive patients in HRQOL in both MCS and PCS Short-Form Health Survey (SF)-36 components was of small magnitude (1). Likewise, in a similar study with 466 participants from Spain, the authors did not find differences in the HRQOL component scores (6). Probably, the fact that patients enrolled in our study had a low percentage of comorbidities and cardiovascular complications may enhance this result. Moreover, arterial hypertension usually does not have symptoms (except the cases of malignant hypertension and hypertensive emergencies and urgencies) and patients with uncomplicated hypertension are not usually alert. In a cross-sectional study that included 3,368 hypertensive patients (23), neither awareness of hypertension nor antihypertensive drug treatment and control of blood pressure appears to influence HRQL. Moreover, approximately 50% of the patients over 50 years suffer from arterial hypertension (5) and thus, this disease is usually considered as an expected result of aging.

In addition, greater levels of depression were found in women and in those with diabetes while Dippers reported lower levels of depression. This is in accordance with several studies (14, 15, 18) while the fact that dippers reported lower levels of depression may be attributed to the fact that dipping status is associated with the quality of sleep and anxiety. Several studies reported that non-dipper patients have significantly higher anxiety and depression scores compared to dipper patients (24). Kayano et al. showed that non dipper patients presented a 4.48-fold higher risk to have an anxiety disorder in confront to dippers (25).

### Limitations

The major limitation of this study is the small sample size. However, we have to underline that this was a single-center study and we performed a comprehensive clinical and laboratory investigation in order to assess total cardiovascular risk of these patients including a comprehensive assessment of target organ damage (full echocardiographic assessment, carotid intima media thickness, etc.). In addition, in this study, the percentage of diabetes, renal impairment, and stroke were relatively low. Increased percentage of these diseases may negatively affect HRQOL results of the total sample. However, as mentioned above, this was a prospective study that included patients from the outpatient hypertension clinic. Intentionally including patients with increased percentage of these diseases may alter the prospective character of the study. Moreover, all self-reported inventories can be easily exaggerated or minimized by the person completing them. Like all questionnaires, the way the instrument is administered can have an effect on the final score. However, this is a common limitation of these studies. Finally, the number of patients with arterial hypertension and diabetes mellitus and/or COPD was relatively low. Thus, more studies with larger sample are needed in order to better assess patients with these characteristics. The total enrollment period was 51 days (from October 1, 2016, to November 21, 2016). Longer period of time may lessen any effect of the season on the patient’s QOL. Finally, bipolar depression was not considered.

## Conclusion

Factors such as gender, age, and diseases such as diabetes mellitus, COPD, renal impairment affect physical and mental health; however, the stage and awareness of arterial hypertension in this study, did not affect these parameters. The fact that arterial hypertension *per se* is not a symptomatic disease may explain these results at least in patients with uncomplicated hypertension.

## Ethics Statement

This study was designed in line with the recommendations of the latest Declaration of Helsinki (2013), the ISPE Guidelines for Good Pharmacoepidemiology Practice, and the ICH Guidelines for Good Clinical Practice, and was approved by the appropriate Ethics Committee (Scientific Councils) of participating hospital departments. All patients gave informed consent prior to their inclusion in the study.

## Author Contributions

All the authors (VK, MK, KK, PS, KS, CT, PN, GP, and DT) contributed in writing the article and approved the final version.

## Conflict of Interest Statement

The authors declare that the research was conducted in the absence of any commercial or financial relationships that could be construed as a potential conflict of interest.
